# Climate anxiety among nursing students: a scoping review

**DOI:** 10.1186/s12912-026-04656-2

**Published:** 2026-04-14

**Authors:** Yaqi Wu, Hejia Wan, Lisha Zhao, Jie Liu, Lulu Zhang, Jing Mao, Haozhe Sun, Zilin Zhao, Xin Wang, Xiaolei Jing

**Affiliations:** 1 School of Nursing (Nursing School of Smart Healthcare Industry), Henan University of Chinese Medicine, Zhengzhou, 450046 Henan People’s Republic of China; 2 Department of Nursing, The First Affiliated Hospital of Henan University of Chinese Medicine, Zhengzhou, 451200 Henan People’s Republic of China; 3 Henan Luoyang Orthopedic-Traumatological Hospital, Zhengzhou, 471002 Henan People’s Republic of China

**Keywords:** Climate anxiety, Nursing students, Scoping review, Nursing education

## Abstract

**Background:**

Climate change is a critical 21st-century global challenge affecting the environment, public health, and social well-being. Climate anxiety is increasingly prevalent among nursing students, who bear a dual burden of personal psychological stress and professional responsibility for addressing climate-related health consequences. However, synthesized evidence on this topic remains limited.

**Aims:**

To comprehensively map the assessment tools, influencing factors, and intervention strategies related to climate anxiety among nursing students, identify key knowledge gaps, and provide evidence-based recommendations for future research and nursing education.

**Design:**

Scoping review conducted following the Arksey and O’Malley framework and reported in accordance with the PRISMA extension for Scoping Reviews (PRISMA-ScR) 2020 guidelines.

**Data sources:**

Seven major electronic databases (PubMed, Cochrane Library, Embase, CINAHL, Web of Science Core Collection, Scopus, and Medline) were searched from database inception to March 1, 2026.

**Methods:**

Literature was screened based on the PCC (Population, Concept, Context) framework, followed by rigorous two-stage independent screening and standardized data charting by two reviewers.

**Results:**

Thirteen studies involving a total of 5,096 nursing students were included. All studies were published between 2024 and 2025, with the majority conducted in Turkey (*n* = 8), followed by Egypt (*n* = 3), Saudi Arabia (*n* = 1), and South Korea (*n* = 1). Findings confirmed a notable prevalence of climate anxiety among nursing students, shaped by multiple interrelated factors including gender, academic year, climate change awareness, intolerance of uncertainty, environmental literacy, and media exposure. Four validated assessment tools were identified. Two intervention studies (one RCT and one quasi-experimental study) suggested that structured educational programs may help mitigate certain dimensions of climate anxiety.

**Conclusions:**

Climate anxiety significantly impacts nursing students’ psychological well-being and professional readiness. Targeted educational initiatives incorporating climate change content and psychological support components are warranted. Future research should address the current geographic and methodological limitations, refine population-specific assessment tools, and rigorously evaluate psychological interventions, including cognitive behavioral approaches.

**Impact:**

This review provides practical guidance for nursing educators and healthcare practitioners, effectively strengthening future healthcare professionals’capacity to address climate-related challenges.

**Public contribution:**

Although there was no direct public or patient involvement, the review’s findings indirectly benefit the public by informing better care. Future research could integrate nursing student feedback to further refine related designs.

**Clinical trial number:**

Not applicable, as this is a scoping review that does not involve clinical trial registration.

## Introduction

Climate change refers to long-term changes in patterns of temperature, precipitation, and wind in the atmosphere, manifested as global temperature rise, droughts, and heavy rainfall [1]. Climate change has been recognized as one of the most severe challenges to global public health in the 21st century [2]. The World Health Organization (WHO) report states that its impact is not merely an environmental crisis, but also involves broader social and psychological crises [3]. Forced migration or displacement caused by climate change, accompanied by uncertainty and re-adaptation processes, increases intergroup conflicts and the incidence of mental illnesses [4]. A series of surveys documented that climate change also brings about various negative emotional experiences, among which anxiety is the most common [5]. Against this backdrop, climate anxiety has emerged as a distinct psychological phenomenon [6]. It is important to note that individuals can experience significant anxiety through media coverage, even without experiencing severe climate change directly [7]. This emotional distress is particularly prominent among global youth populations [8]. A large-scale international survey revealed that up to 84% of adolescents reported moderate to severe climate anxiety, with 45% indicating that it has already impacted their daily lives [9]. This psychological response not only affects individual emotional states but may also exert profound impacts on cognition, behavior, and social functioning [10].

**Fig. 1:**
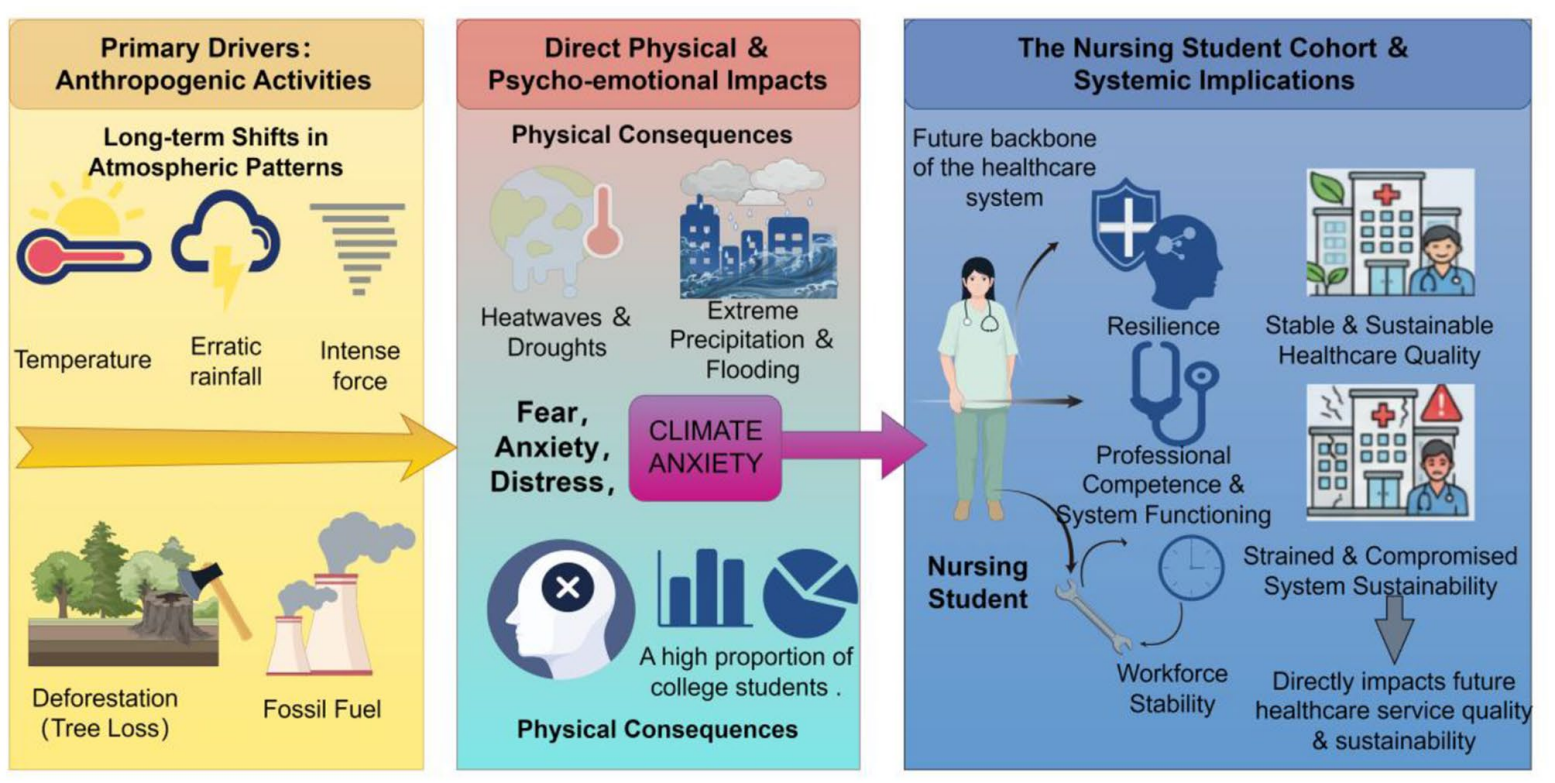
The impact pathway: climate change, climate anxiety, and nursing students’ mental health

Among the affected youth, nursing students constitute a critical and unique subgroup. As shown in Fig. 1, climate change impacts not only the environment but also nursing students’ mental health. First, as members of the global youth population, they personally experience the pervasive psychological stress caused by the climate crisis. Second, and more uniquely, as future healthcare professionals, they are about to enter an industry that must directly address the health consequences of climate change—from heatstroke and surges in infectious diseases to post-disaster mental health crises [11]. This dual identity may lead to significant role conflict: personal climate anxiety and concerns about inadequate professional preparedness, professional ethical responsibility, and a sense of powerlessness when facing large systemic problems. However, despite the increasing importance of this issue, existing research remains fragmented. Although studies on climate anxiety in the general population have been growing [12, 13], and discussions on sustainable development in nursing education already exist, evidence specifically focusing on nursing students’ experiences of climate anxiety, its influencing factors, and professional consequences is still lacking systematic integration and review [14–16]. In recent years, the scoping review methodology has proven highly effective in nursing science for comprehensively mapping complex cognitive concepts and professional experiences within specific nursing populations [17]. Therefore, conducting a scoping review in this area is crucial for mapping the knowledge landscape, clarifying core concepts, guiding future research, and shaping educational strategies and support systems. Thus, this study systematically reviews the research progress on climate anxiety among nursing students using the JBI scoping review guidelines as the methodological framework. It aims to provide scientific references for educational strategies and mental health support regarding climate anxiety among nursing students.

## Methods

### Design

A scoping review was conducted following the framework proposed by Arksey and O’Malley [18]. They typically progress in five key stages (1) identifying the research question; (2) identifying the relevant studies; (3) selecting the studies; (4) charting the data and (5) collating, summarising and reporting the results. The scoping review was conducted according to Joanna Briggs Institute (JBI) guidelines [19], utilizing the Preferred Reporting Items for Systematic Reviews and Meta-Analyses extension for Scoping Reviews (PRISMA-ScR) 2020 framework to guide the reporting of this scoping review [20].

### Identifying the research question

The research questions of this study are: (1) What is the current status of climate anxiety among nursing students? (2) What are the assessment tools for climate anxiety among nursing students? (3) What are the main influencing factors of climate anxiety among nursing students? (4) What are the strategies and interventions for coping with climate anxiety?

### Identifying relevant studies

Inclusion criteria were determined based on the “PCC” framework: (1) Participants (P): Nursing students. (2) Concepts (C): Climate anxiety, climate change anxiety. (3) Context (C): Nursing education, nursing training, nursing curriculum. Exclusion criteria: (1) Full text not accessible; (2) Non-English articles; (3) Duplicate publications.

### Study selection

The databases searched include PubMed, the Cochrane Library, Embase, CINAHL, the Web of Science Core Collection, Scopus and Medline. The search timeframe was from the establishment of the database until March 1, 2026. A combination of subject headings and free terms was used for the search, with both subject headings and free terms selected from the Medical Subject Headings (MeSH). For the English databases, using PubMed as an example, a combination of MeSH terms, free terms, and Boolean operators was used for the search. The search strategy was: ((“Students, Nursing“[Mesh]) OR “Nursing Student“[Title/Abstract] OR “Nursing Students“[Title/Abstract] OR “Pupil Nurse“[Title/Abstract] OR “Pupil Nurses“[Title/Abstract]) AND ((“Climate Change“[Mesh]) OR “Climate Change“[Title/Abstract] OR “climate anxiety“[Title/Abstract] OR “eco-anxiety“[Title/Abstract] OR “global warming“[Title/Abstract] OR “sea level rise“[Title/Abstract]) AND ((“Anxiety“[Mesh]) OR Anxiety[Title/Abstract] OR Nervousness[Title/Abstract] OR Hypervigilance[Title/Abstract] OR “Social Anxiety“[Title/Abstract] OR Anxiousness[Title/Abstract]).

To ensure maximum methodological transparency and reproducibility, the detailed search strategies for all databases and the data extraction forms have been deposited in an anonymized Open Science Framework (OSF) repository, available at: https://osf.io/vazds/overview?view_only=c58d6730711946fba5495ab4ec8f5a36.

### Charting the data

**Fig. 2:**
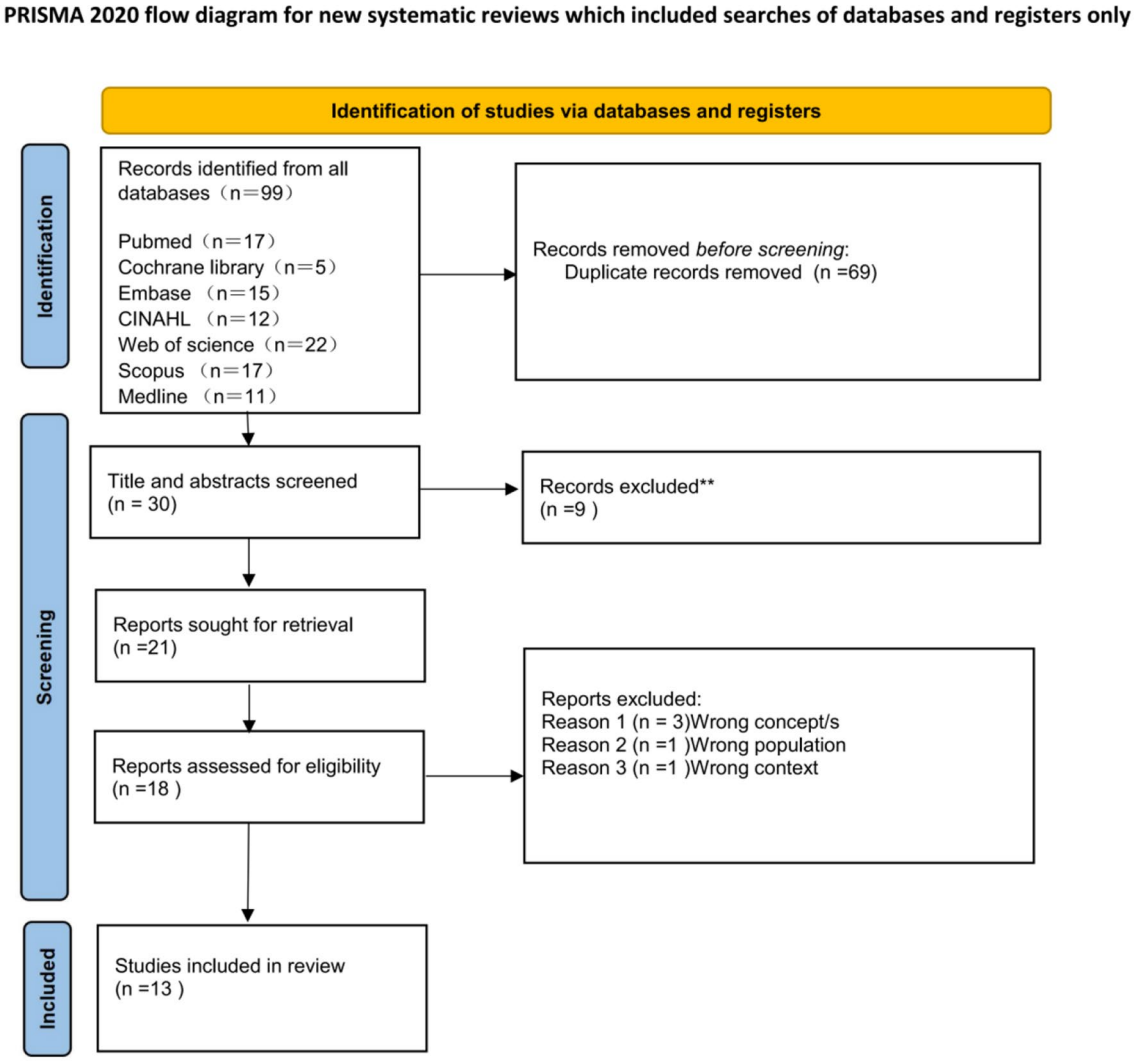
The search and selection process

The retrieved literature was imported into NoteExpress for organization, and duplicate articles were removed. Two graduate students trained in evidence-based methods independently reviewed the titles and abstracts for initial screening, and then read the full text of potentially eligible articles for a second screening, comparing the results. In case of disagreement, a discussion with a third researcher was held to finalize the inclusion of articles meeting the criteria. The data extraction included information on the author, country, publication year, study subjects, background, objectives, methods, research tools for climate anxiety, current status, and influencing factors. The search and selection process is illustrated in Fig. 2, outlining how the literature was systematically reviewed.

### Collating, summarizing and reporting the results

We systematically reviewed all relevant studies included in the analysis, focusing on the research questions, and provided a comprehensive narrative summary of the literature on this topic. The research findings are presented and categorized into four key themes: (1) What is the current status of climate anxiety among nursing students? (2) What assessment tools are utilized to measure climate anxiety among nursing students, and what are their methodological applicability and limitations? (3) What are the main influencing factors of climate anxiety among nursing students? (4) What are the strategies and interventions for coping with climate anxiety?

## Results

A preliminary search identified a total of 99 records from all databases. After removing 69 duplicate records, 30 articles remained for title and abstract screening. During this phase, 9 records were excluded, leaving 21 reports sought for retrieval. Subsequently, 18 reports were assessed for full-text eligibility. Full-text screening led to the exclusion of 5 reports for the following reasons: wrong concepts (*n* = 3), wrong population (*n* = 1), and wrong context (*n* = 1). Finally, 13 studies were included in the review.

### Characteristics of included studies

A total of 13 studies focusing on climate anxiety or eco-anxiety among nursing students were included in this scoping review. The characteristics of the studies included in this review are summarized in Table 1. Geographically, the majority of the studies were conducted in Turkey (*n* = 8) [14–16, 21–25], followed by Egypt (*n* = 3) [26–28], Saudi Arabia (*n* = 1) [29], and South Korea (*n* = 1) [24].

The included studies predominantly employed a variety of cross-sectional designs (*n* = 11), alongside one quasi-experimental study and one randomized controlled trial (RCT). The methodological approaches ranged from descriptive and correlational surveys to more complex analytical models (e.g., moderation analysis) and rigorous experimental designs. All studies were carried out in undergraduate nursing programs across universities, with the majority being single-center studies. Furthermore, all studies were published recently, in 2024 and 2025, indicating a rapid and growing interest in researching the relationship between climate change and psychological distress (such as climate anxiety and eco-anxiety) among nursing students.

A precise total of 5,096 nursing students were included across the 13 selected studies. The sample sizes varied considerably, ranging from 117 to 620 participants per study. Demographically, the vast majority of the participants were female, reflecting the gender distribution typical in nursing education. The mean age of the participants generally ranged from 20 to 23 years, with most studies focusing on undergraduate nursing students across their first to fourth years of study. Notably, the study conducted in Saudi Arabia reported a median participant age of 23 years.

Regarding methodological and ethical procedures, all 13 included studies reported obtaining formal ethical approval from their respective institutional review boards and explicitly emphasized the acquisition of informed consent from all participants. Data were collected primarily through online questionnaires, while the remaining studies utilized traditional paper-based surveys. Across all investigations, researchers maintained a rigorous commitment to voluntary participation, data confidentiality, and anonymity, thereby ensuring the ethical integrity of the data collection process.

**Table 1:** Basic features of the included literature

Title	Authors	Year	Country	Aim	Method	Sample Size/Tool
Is climate change awareness a predictor of anxiety among nursing students?: A cross-sectional study [14]	Çelik Eren D, Kabataş Yıldız M	2024	Türkiye	To determine whether nursing students’ global climate change awareness predicts their climate change anxiety	Descriptive cross-sectional study (online questionnaire)	419/CCAS
Is awareness of climate change a predictor of eco-anxiety? Research within the scope of nursing students [15]	Baykara Mat ST et al.	2024	Türkiye	To analyze whether climate change awareness predicts eco-anxiety among nursing students	Cross-sectional correlational study	390/Eco-Anxiety Scale
Nature-friendly hands: the relationship between nursing students’ climate change anxiety, intolerance of uncertainty, and anxiety about the future [21]	Güler KG et al.	2024	Türkiye	To examine the relationships among climate change anxiety, intolerance of uncertainty, and future anxiety in nursing students	Correlational survey study	321/CCAS
Nursing students’ mental health: how does eco-anxiety effect? [22]	Er S et al.	2024	Türkiye	To analyze the effect of eco-anxiety on nursing students’ mental health	Descriptive cross-sectional correlational study	609/Eco-Anxiety Scale
Relationship between nursing students’ global climate change awareness, climate change anxiety and sustainability attitudes in nursing: a descriptive and cross-sectional study [16]	İlaslan N et al.	2024	Türkiye	To examine the relationships among global climate change awareness, climate change anxiety, and sustainability attitudes	Descriptive cross-sectional study	289/CCAS
Climate Change Related Health Problems and Awareness of Nursing Students: A Cross-Sectional Study [23]	Çetin, A.Ö.et al.	2025	Türkiye	To examine nursing students’ levels of knowledge, awareness, motivation, anxiety and behavior regarding climate-change-related health problems and to identify influencing factors.	Single-center descriptive cross-sectional survey	386/CHANT
Effect of climate change and health course on global warming knowledge and attitudes, environmental literacy, and eco-anxiety level of nursing students: a quasi-experimental study [24]	Çolak M et al.	2025	Türkiye	To examine the effect of a “Climate Change and Health” course on global warming knowledge, attitudes, environmental literacy, and eco-anxiety levels	Quasi-experimental study	117/Eco-Anxiety Scale
The psychological impact of climate change: exploring the link between media induced indirect trauma and climate anxiety [25]	Ediz Ç, Yanık D, Brimoglu Okuyan C, Uzun S	2025	Türkiye	To determine the relationship between indirect trauma from media exposure to climate change events and climate anxiety among nursing students	Descriptive cross-sectional study (online survey)	580/CCAS
Future nurses in a changing climate: exploring the relationship between environmental literacy and climate anxiety [26]	Amin SM et al.	2024	Egypt	To explore the relationship between environmental literacy and climate anxiety among future nurses	Cross-sectional survey	620/CCAS
Video-based climate change program boosts eco-cognizance, emotional response and self-efficacy in rural nursing students: randomised controlled trial [27]	Eweida RS et al.	2025	Egypt	To investigate the effect of a video-based climate change program on eco-cognizance, emotional response, and environmental self-efficacy among rural nursing students	Randomised controlled trial (RCT)	140 (70 intervention, 70 control)/CCAS
Climate change related depression, anxiety and stress among faculty of nursing students at Assiut University [28]	Mohamed MA et al.	2025	Egypt	To assess levels of depression, anxiety, and stress related to climate change among nursing students	Descriptive cross-sectional study	330/DASS-21
Nursing students’ global warming knowledge and sustainability attitudes: the moderating role of eco-anxiety [29]	Berdida DJE et al.	2025	Saudi Arabia	To investigate the moderating role of eco-anxiety between global warming knowledge and sustainability attitudes	Cross-sectional moderation analysis	561/Eco-Anxiety Scale
Effects of climate change anxiety and environmental sustainability attitudes on pro-environmental behaviors among nursing students: a cross-sectional study [24]	Lee Y et al.	2025	South Korea	To investigate the effects of climate change anxiety and environmental sustainability attitudes on pro-environmental behaviors	Cross-sectional study	334/CCAS

### Assessment tools for climate anxiety among nursing students

To quantify the psychological burden of climate change among nursing students, the included studies primarily utilized four distinct measurement instruments: the Climate Change Anxiety Scale (CCAS) [30], the Hogg Eco-Anxiety Scale (HEAS) [31], the Anxiety Subscale of the Climate, Health, and Nursing Tool (CHANT) [32], and a contextually modified version of the Generalized Anxiety Disorder Scale-7 (GAD-7) [33]. The comprehensive psychometric properties of these tools—including their underlying dimensions, item counts, scoring metrics, internal consistency (Cronbach’s α), and specific validation status within nursing student populations—are systematically detailed in Table 2.

An appraisal of these four instruments reveals several substantive methodological concerns with direct implications for the interpretation of findings across included studies. First, with the sole exception of the CHANT [32], none of the identified instruments were originally developed or validated for use in nursing student populations. The CCAS [30] and HEAS [31] were constructed and psychometrically evaluated in general community samples, whereas the GAD-7 [33] was designed as a brief clinical screener for generalized anxiety disorder and was subsequently adapted in one included study to attribute symptom content to climate-related stressors — a modification that has not undergone systematic psychometric scrutiny. The application of these instruments to nursing students therefore raises legitimate concerns regarding construct validity and measurement equivalence [23, 34]. Nursing students occupy a conceptually distinct position relative to the general public: their experience of climate anxiety is embedded within an emerging professional identity that encompasses anticipated clinical responsibility for managing climate-attributable health outcomes, ethical obligations toward environmental advocacy, and an acute awareness of potential occupational unpreparedness. These professionally situated dimensions of climate-related psychological distress are unlikely to be adequately captured by tools developed without reference to this population’s unique contextual characteristics.

Second, the concurrent use of four instruments with divergent theoretical underpinnings, dimensional structures, and response formats across the thirteen included studies substantially constrains cross-study comparability. Each instrument operationalizes climate anxiety through a distinct conceptual lens — ranging from cognitive-emotional and functional impairment (CCAS) [30] to affective, ruminative, and behavioral symptom clusters (HEAS) [31] and generalized anxiety phenomenology reframed in a climate context (modified GAD-7) [33]—such that prevalence estimates, factor associations, and intervention outcomes derived from different tools cannot be meaningfully aggregated or directly compared. This measurement heterogeneity constitutes a fundamental impediment to cumulative evidence synthesis in this emerging field [35].

Third, although the CHANT [32, 36] represents the most contextually appropriate instrument identified in this review — having been developed specifically for nurses and nursing students and demonstrating strong internal consistency (Cronbach’s α = 0.95), its anxiety subscale encompasses only five items, a constraint that may limit its capacity to capture the full multidimensional structure of climate anxiety as a psychological construct. Moreover, evidence supporting the cross-cultural measurement invariance of the CHANT across linguistically and culturally diverse nursing student populations remains sparse, thereby limiting its applicability in international research contexts.

Collectively, these observations delineate a critical gap in the current evidence base: the field lacks a psychometrically rigorous, multidimensional, and nursing student specific instrument for assessing climate anxiety that adequately reflects the distinctive professional and educational context of this population [35]. The development, validation, and cross-cultural adaptation of such a tool should be regarded as a methodological priority for future research in this domain.

**Table 2:** Characteristics of climate anxiety measurement instruments

Tool	Developer (s)/Year	Main Measurement Dimensions	Number of Items	Scoring Standard	Cronbach’s α	Validation Status in Nursing/Nursing Student Populations
Climate Change Anxiety Scale (CCAS) [30]	Clayton & Karazsia / 2020	Cognitive-emotional impairment Functional impairment	13	5-point Likert scale (1 = Never to 5 = Almost always) Higher total scores indicate greater climate change anxiety	α ≈ 0.90–0.93;	Not developed for nursing populations; potential applicability in nursing students, but no large-scale dedicated validation in this group
Hogg Eco-Anxiety Scale (HEAS) [31]	Hogg et al. / 2021	Affective symptoms Rumination Behavioural symptoms Anxiety about personal impact	13	4-point frequency scale (0 = Not at all to 3 = Nearly every day) Higher subscale and total scores indicate greater eco-anxiety	α = 0.86–0.92	Not developed for nursing populations; general/cross-age validation, no dedicated nursing student validation
Climate, Health, and Nursing Tool (CHANT)-Anxiety Subscale [32]	Schenk et al. / 2019 (initial development; CFA validation 2023)	Anxiety subscale: Concern regarding negative impacts of climate change on health (Full tool includes broader factors: Awareness, Motivation, Concern, Behaviors)	Anxiety subscale: 5 items	5-point Likert scale Higher scores indicate greater climate-related concern/anxiety	α = 0.95	Specifically developed for nurses and nursing students (later extended to health professionals); validated in nurse/nursing student samples with strong reliability
Generalized Anxiety Disorder Scale-7 (GAD-7) [33]	Spitzer et al. / 2006	Generalized anxiety symptoms	7	0–3 Likert (0 = Not at all → 3 = Nearly every day)	α = 0.92	Not originally developed for climate anxiety; Arabic/Egyptian versions validated with high reliability (α > 0.90) in nursing/medical students; modified here to link symptoms to climate anxiety

### Influencing factors of climate anxiety among nursing students

#### Demographic characteristics

Demographic characteristics have been consistently identified as significant correlates of climate anxiety among nursing students. With respect to gender, female students demonstrate significantly elevated climate anxiety relative to their male counterparts across multiple investigations [16, 26]. This pattern is theoretically attributable to the synergistic influence of gender-specific socialization processes and neuroendocrine mechanisms, including heightened amygdala reactivity and estrogen-mediated facilitation of negative memory consolidation [37, 38], alongside a greater propensity to extend empathic concern beyond personal risk to encompass broader domains such as ecosystems and future generations, collectively amplifying overall affective burden. Academic year also emerges as a meaningful correlate of psychological vulnerability. Third-year nursing students, in particular, have been found to exhibit markedly higher levels of climate anxiety and associated psychological distress relative to other cohorts [16]. This finding may reflect the psychological consequences of mid-program curricular exposure: having acquired a partial but not yet consolidated understanding of climate-related health impacts, these students may be particularly susceptible to catastrophic appraisal and intolerance of uncertainty, a cognitive profile consistent with incomplete knowledge integration. Corroborating this pattern, students aged 20 years or younger have been found to report significantly greater climate-associated psychological distress than their older peers [39], a finding that aligns with their comparatively lower climate change knowledge and less developed attitudinal frameworks.

#### Climate change awareness

Climate change awareness refers to an individual’s level of understanding of climate change phenomena, including knowledge of the causes, consequences, and its impacts on health, the environment, and society. A study by Çelik Eren et al. [14] found that nursing students’ awareness of climate change significantly affects their level of climate anxiety, with greater knowledge about climate change correlating with higher anxiety levels. This finding is consistent with the research by Ilaslan et al. [16] and Baykara Mat et al. [15] When nursing students become aware of the threats and severity of climate change, they experience stronger anxiety and fear. Ilaslan et al. [16] found that, within the nursing student population, this relevant information often comes from social media. Media coverage of climate change is frequent and often presented in a negative light, such as extreme weather events and ecological disasters [40], which may exacerbate students’ emotional distress. This suggests that nursing educators should utilize evidence-based scientific resources in the curriculum to enhance nursing students’ accurate and evidence-based understanding of climate change, guide them to critically evaluate information from social media and news reports [16], and apply a positive psychology perspective to interpret the relevant information rationally, reducing anxiety caused by information asymmetry.

#### Intolerance of uncertainty

Intolerance of uncertainty refers to an individual’s cognitive, emotional, and behavioral responses to uncertain events in life [41]. Individuals with high intolerance of uncertainty are more likely to perceive uncertainty as a threat and distressing, often attempting to avoid these uncertain situations [42]. A study by Gülırmak et al. [21] found a significant positive correlation between nursing students’ intolerance of uncertainty and climate change anxiety. This may be because climate change itself is a global issue full of uncertainty, with the impacts and consequences of climate change being difficult to predict, and climate science and policies continuously evolving. Therefore, for nursing students with high intolerance of uncertainty, the uncertainty surrounding climate change makes them feel more uneasy when faced with unpredictable environmental changes in the future, thereby exacerbating their anxiety. Furthermore, the subdimension of intolerance of uncertainty, “anticipatory anxiety,” is positively correlated with the subdimension of climate change anxiety, “behavioral engagement,” indicating that nursing students tend to convert their concerns about climate change into action, explaining their motivation to act in response to climate anxiety. Studies have shown that there is a positive relationship between anxiety and hope, and a link between hope and action, thus, taking action is considered one of the predictors of good psychological health [43]. In conclusion, nursing educators can implement psychological support programs to help students manage intolerance of uncertainty and future-oriented anxiety, and develop interactive educational activities on climate change and sustainability, providing nursing students with opportunities to engage in climate change-related research.

#### Future anxiety

Future anxiety is the persistent worry and negative expectation about impending threats, losses, or uncertainties, accompanied by physiological activation and functional impairment. In this study, future anxiety specifically refers to the ongoing fear of future quality of life and security caused by concerns about the deterioration of the Earth’s ecosystems, increased extreme events, and worsened living conditions for future generations. Gulirmak et al. [21] found that reducing future anxiety can partially mitigate climate anxiety. This is because future anxiety has a significant positive predictive effect on climate anxiety through two pathways: catastrophic rumination and emotional labeling. Therefore, educational interventions (such as future scenario construction, controllable action training, and enhancing hope) help reduce future anxiety, thereby alleviating individual climate anxiety. Therefore, nursing education should implement educational interventions to help nursing students reduce future anxiety [44] and provide relevant emotional regulation strategies to effectively alleviate climate anxiety.

#### Environmental literacy

Environmental literacy refers to the knowledge, skills, attitudes, and behaviors that enable an individual to make informed environmental decisions and take actions in ways that promote sustainability [45]. Environmental literacy in nursing is crucial as it empowers nurses to advocate for sustainable practices and helps patients understand environmental health risks, thereby mitigating the impacts of climate change [46]. A study by Amin et al. [26] found a significant positive correlation between nursing students’ environmental literacy and climate anxiety, meaning that the more nursing students know about environmental issues, the more likely they are to experience climate change anxiety. This correlation reflects that while environmental literacy provides the knowledge and skills needed to address climate change, it may also exacerbate individuals’ concerns and anxieties about the consequences of climate change. Among the subdimensions of environmental literacy, environmental awareness was not correlated with climate anxiety, suggesting that although nursing students are concerned about environmental issues, they may not fully understand the broad impacts of climate change. Similarly, environmental anxiety is significantly positively correlated with climate anxiety, indicating that when individuals have strong emotional reactions to environmental issues (such as pollution or ecological destruction), their concerns about climate change are often more intense. Furthermore, surveys have shown that nursing students generally possess high environmental literacy, which may be related to the emphasis in basic nursing education on the relationship between humans, the environment, and health [47]. In conclusion, the results of this study emphasize the importance of integrating comprehensive environmental literacy into nursing education programs, including providing nursing students with the fundamental knowledge, skills, and attitudes required to address climate change and its impacts on public health; additionally, environmental modules should be included in nursing curricula, and mental health support services, such as stress management and mindfulness training, should be provided to help alleviate nursing students’ climate anxiety in a timely manner [24].

#### Media exposure and news consumption patterns

Media exposure to climate change refers to the process by which individuals encounter, perceive, and process climate-related information through various digital and traditional media channels—including internet platforms, social media applications, and television broadcasts—without direct personal experience of climate-related events [48]. In this study [25], media exposure was operationalized along two dimensions: the platform type through which climate news is primarily accessed, and the daily duration of engagement with climate-related content. Ediz et al. [25] found that both dimensions significantly predicted climate anxiety levels among nursing students. Regarding exposure duration, students who spent more time engaging with climate-related news reported higher levels of climate anxiety. Regarding platform type, students who primarily accessed news via internet-based platforms demonstrated significantly higher climate anxiety compared to those who relied on newspapers or radio. This may be explained by prolonged engagement with climate-related content overwhelming individuals’ cognitive regulatory capacity, resulting in sustained threat appraisal and feelings of helplessness [49]. Additionally, internet-based platforms, driven by engagement-maximizing algorithms, tend to prioritize emotionally charged and catastrophic climate narratives, which may amplify perceived environmental threats compared to traditional media [50]. Furthermore, repeated exposure to distressing climate content may trigger vicarious traumatization [51, 52], a process particularly relevant for nursing students whose professional socialization emphasizes empathy, rendering them more susceptible to affective contagion through media consumption [22]. Therefore, nursing education should incorporate media literacy training to help nursing students critically evaluate climate-related content, regulate media consumption habits, and develop adaptive emotional coping strategies, so as to effectively mitigate media-induced climate anxiety [53].

## Implications for nursing education

### Integrating climate change-related content into nursing education curricula

**Fig. 3:**
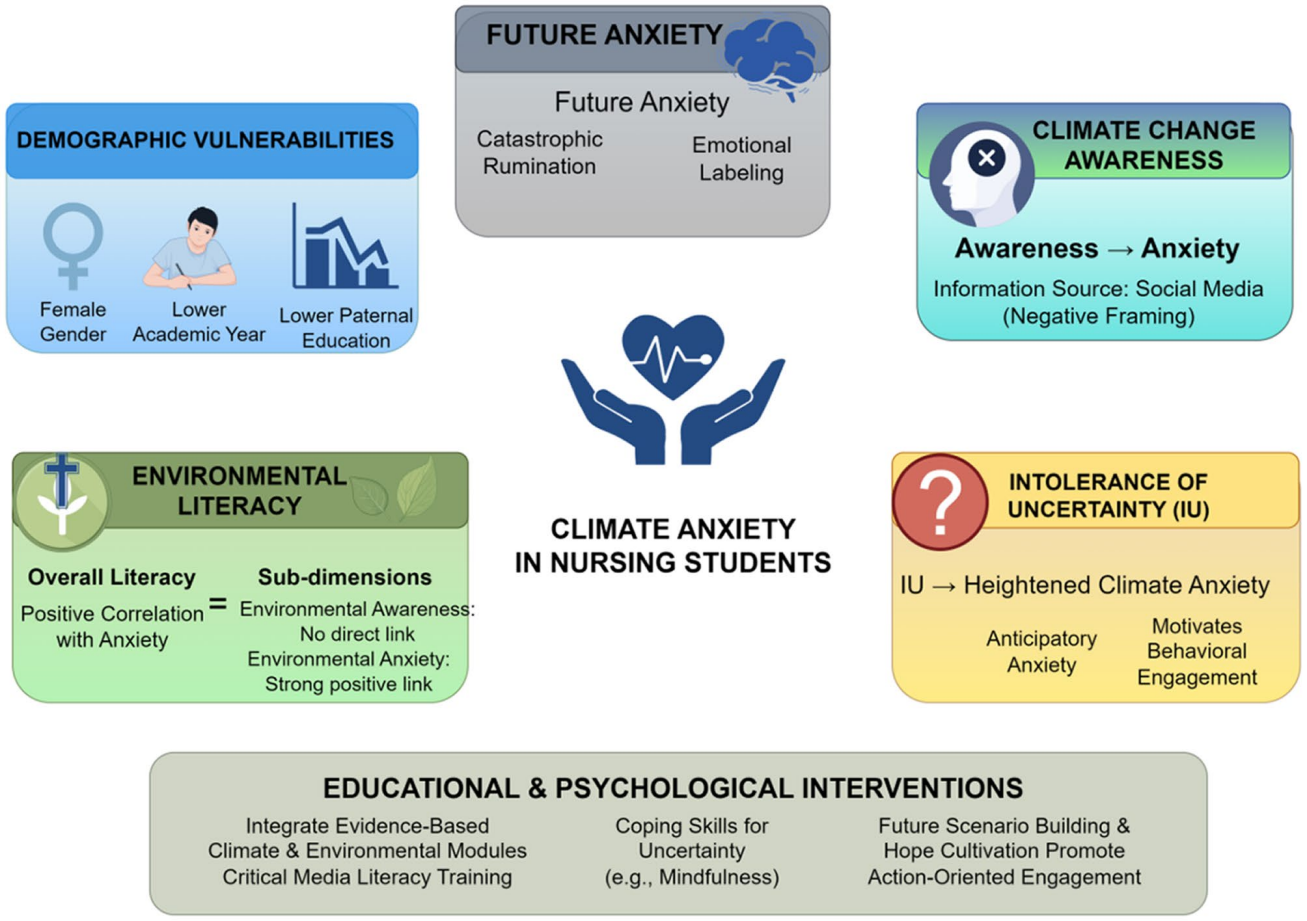
Factors influencing climate anxiety among nursing students

As shown in Fig. 3, multiple factors influence the levels of climate anxiety in nursing students. Although the connection between climate change and health has been recognized, many nursing curricula have not systematically incorporated this content [24]. Portela et al. [54] pointed out that nursing education should first cultivate students’ awareness of climate change. Ilaslan et al. [16] found that higher climate change awareness was associated with climate anxiety levels and sustainability attitudes, suggesting that education may help students better manage anxiety and convert it into constructive action. Therefore, nursing education curricula should be reformed to integrate knowledge of climate change and health into all aspects of nursing education [39]. By regularly updating course content, conducting interdisciplinary teaching, and providing practical training, nursing students’ ability to respond to climate change should be enhanced, preparing them to better address the health challenges posed by climate change in clinical practice [55]. As future health care providers, nursing students must possess sufficient knowledge of climate change [39]. Research shows that nursing students with higher awareness of climate change often experience higher levels of climate anxiety, suggesting that nursing education should strengthen courses on the relationship between climate change and health, helping students understand the specific impact of climate change on health [14–16]. The curriculum should also include basic knowledge such as the scientific principles and mechanisms of climate change, the specific impacts of climate change on public health, such as respiratory diseases related to air pollution and diarrhea caused by floods via contaminated drinking water, environmental awareness, environmental protection literacy, and sustainable nursing practices [54, 56, 57]. Real-life cases of weather changes, such as smog, heavy rain, and other extreme weather events, should be incorporated into nursing scenario-based simulation teaching to help students understand the specific health impacts of climate change and the role of nurses in addressing these impacts [58].

### Providing mental health support for nursing students

#### Digital media-based educational interventions

Evidence from randomized controlled trials suggests that structured digital media-based educational interventions represent a promising and scalable strategy for alleviating climate change-related anxiety among nursing students. Eweida et al. [27] demonstrated that a video-based climate change program delivered to nursing students in rural communities produced significantly lower levels of cognitive impairment attributable to climate change anxiety in the intervention group relative to controls, accompanied by large effect sizes in improvements in climate change perception and environmental self-efficacy. This finding underscores the mechanistic role of self-efficacy enhancement in anxiety mitigation: when nursing students acquire evidence-based knowledge of climate phenomena and develop a confident sense of agency in responding to environmental challenges, maladaptive cognitive responses to climate-related stressors are correspondingly attenuated. The use of video-based modalities is particularly well-suited to resource-limited or geographically dispersed educational settings, offering flexible, repeatable, and visually engaging content that facilitates deeper affective engagement compared with passive informational materials such as pamphlets or flyers. Future interventions should therefore incorporate structured multimedia curricula that explicitly target self-efficacy alongside knowledge acquisition, and should employ validated instruments such as the Climate Change Anxiety Scale to monitor anxiety trajectories throughout program delivery.

#### Formal curriculum integration with psychological support components

Beyond discrete educational programs, the formal integration of climate change content into nursing curricula represents a systematic institutional approach with documented effects on students’ psychological responses to environmental stressors. Çolak et al. [24] conducted a quasi-experimental study among 117 undergraduate nursing students enrolled in a dedicated “Climate Change and Health” course, finding that course completion was associated with statistically significant increases in both global warming knowledge and attitude scores, as well as in eco-anxiety total scores and behavioral symptom subscale scores. Notably, the observed elevation in eco-anxiety following the curriculum-based intervention warrants careful pedagogical consideration: heightened anxiety may reflect an adaptive, awareness-driven emotional response rather than a pathological state, and educators must therefore complement knowledge-focused instruction with structured psychological coping strategies, including guided reflection on action-oriented responses, promotion of collective efficacy, and facilitated discussion of emotional responses to climate information. The absence of statistically significant changes in environmental literacy scores further indicates that knowledge acquisition alone is insufficient to foster comprehensive ecological competence, and that affective and behavioral dimensions of learning must be deliberately addressed. Accordingly, nursing educators are advised to design climate-focused curricula that integrate psychosocial support components, such as resilience-building modules, peer-facilitated support groups, and targeted mindfulness-based strategies—to ensure that increased climate awareness translates into constructive engagement rather than paralyzing distress.

#### Potential psychological interventions for climate anxiety in nursing students

Several psychological interventions may prove beneficial in addressing climate anxiety among nursing students [59]. Cognitive behavioral therapy (CBT) offers structured techniques, including cognitive restructuring and behavioral experimentation—that help students identify, examine, and modify maladaptive beliefs related to climate change, such as catastrophic appraisals of natural disasters or climate-related occupational concerns, thereby fostering a more accurate perception of environmental risk and a greater sense of personal agency. Acceptance and Commitment Therapy (ACT), through its six core processes of mindfulness, acceptance, cognitive diffusion, self-as-context, values clarification, and committed action, promotes psychological flexibility and encourages students to acknowledge climate-related distress while engaging in value-driven, adaptive behaviors rather than avoidance. It must be acknowledged, however, that to date no published studies have specifically evaluated the effectiveness of CBT, ACT, or mindfulness-based interventions for climate anxiety in nursing students; these approaches therefore remain theoretical possibilities rather than evidence-based recommendations, and their applicability to this population warrants rigorous empirical investigation in future research.

### Future research directions

Beyond the individual and educational factors identified in this review, future research should consider the potential role of clinical environment complexity in shaping nursing students’ psychological responses to climate-related stressors. Recent nursing research has demonstrated that clinical complexity in hospital settings can be systematically measured and stratified using standardized nursing data [60]. Although such work does not address climate anxiety directly, it highlights that nursing students’ exposure to high-acuity, high-risk clinical environments varies substantially across settings. It is plausible that students training in more complex clinical contexts may experience heightened psychological vulnerability, including greater susceptibility to climate-related distress, given the compounding demands of managing both acute patient care and broader environmental concerns. Integrating measures of clinical complexity into future studies on nursing student climate anxiety may therefore offer a novel and productive line of inquiry, potentially revealing how workplace context moderates the development and expression of climate anxiety during professional training.

## Limitations

Several limitations of this scoping review should be acknowledged. First, the included studies exhibit substantial geographic concentration, with eight of the thirteen studies (62%) conducted in Turkey, three in Egypt, one in South Korea and one in Saudi Arabia, while no studies from North America, Western Europe, or Australia were identified. This regional imbalance may limit the generalizability of findings, as cultural, educational, and healthcare system differences may substantially influence the nature and magnitude of climate anxiety among nursing students across different contexts. Second, the methodological homogeneity of included studies constitutes a notable limitation. Eleven of thirteen studies employed cross-sectional designs, which preclude causal inference and limit the ability to evaluate temporal changes in climate anxiety. Longitudinal and experimental study designs are needed to better understand the developmental trajectory of climate anxiety and the sustained effects of interventions. Third, consistent with standard scoping review methodology, formal critical appraisal of included studies was not conducted. Consequently, the methodological quality of individual studies varies, and findings should be interpreted with caution, as the evidence base includes studies of differing rigor. Readers should note that cross-sectional self-report data are particularly susceptible to social desirability bias and response bias. Fourth, regarding assessment tools, most instruments used in the included studies were originally developed for general or non-nursing populations. With the exception of the CHANT, none of the identified tools have undergone large-scale dedicated validation in nursing student samples, which raises questions about measurement equivalence and construct validity in this specific population. Fifth, the intervention evidence base is extremely limited. Only two studies evaluated structured interventions, and recommendations regarding cognitive behavioral therapy (CBT), acceptance and commitment therapy (ACT), and mindfulness-based strategies discussed in the implications section are derived from the broader mental health literature rather than from studies conducted within the nursing student population. These approaches should therefore be regarded as theoretical possibilities requiring rigorous empirical investigation, rather than established evidence-based recommendations.

## Conclusion

In summary, this review highlights the growing concern of climate anxiety among nursing students and underscores the importance of integrating climate change education and mental health support into nursing curricula. Addressing climate anxiety is not only essential for the well-being of nursing students but also for the preparation of future healthcare professionals to effectively address the health impacts of climate change.

## Relevance for clinical practice

This review provides actionable insights for clinical nurses and nurse educators to address climate anxiety in future healthcare professionals. It identifies validated assessment tools and modifiable influencing factors to guide early identification of at-risk nursing students. By integrating climate change content and mental health interventions into clinical training, practitioners can enhance students’ psychological resilience and prepare them to manage climate-related health challenges in clinical settings.

## Data Availability

The data that support the findings of this study are available from the corresponding author upon reasonable request.
